# Mathematical Models of the Effect of Glucagon on Glycemia in Individuals With Type 2 Diabetes Treated With Dapagliflozin

**DOI:** 10.1210/jendso/bvae067

**Published:** 2024-04-08

**Authors:** Tomoko Yamada, Hikaru Sugimoto, Ken-ichi Hironaka, Yasuko Morita, Hiroshi Miura, Natsu Otowa-Suematsu, Yuko Okada, Yushi Hirota, Kazuhiko Sakaguchi, Shinya Kuroda, Wataru Ogawa

**Affiliations:** Division of Diabetes and Endocrinology, Department of Internal Medicine, Kobe University Graduate School of Medicine, Hyogo 650-0017, Japan; Department of Biochemistry and Molecular Biology, Graduate School of Medicine, The University of Tokyo, Tokyo 113-0033, Japan; Department of Biological Sciences, Graduate School of Science, The University of Tokyo, Tokyo 113-0033, Japan; Division of Diabetes and Endocrinology, Department of Internal Medicine, Kobe University Graduate School of Medicine, Hyogo 650-0017, Japan; Department of Diabetes and Endocrinology, Takatsuki General Hospital, Takatsuki, Osaka 569-1192, Japan; Division of Diabetes and Endocrinology, Department of Internal Medicine, Kobe University Graduate School of Medicine, Hyogo 650-0017, Japan; Department of Diabetes and Endocrinology, Kagayaki Diabetes and Endocrinology Clinic, Hyogo 650-0001, Japan; Division of Diabetes and Endocrinology, Department of Internal Medicine, Kobe University Graduate School of Medicine, Hyogo 650-0017, Japan; Division of Diabetes and Endocrinology, Department of Internal Medicine, Kobe University Graduate School of Medicine, Hyogo 650-0017, Japan; Division of General Medicine, Department of Internal Medicine, Kobe University Graduate School of Medicine, Hyogo 650-0017, Japan; Department of Biochemistry and Molecular Biology, Graduate School of Medicine, The University of Tokyo, Tokyo 113-0033, Japan; Department of Biological Sciences, Graduate School of Science, The University of Tokyo, Tokyo 113-0033, Japan; Division of Diabetes and Endocrinology, Department of Internal Medicine, Kobe University Graduate School of Medicine, Hyogo 650-0017, Japan

**Keywords:** glucagon, mathematical model, production index/clearance, SGLT2 inhibitor, type 2 diabetes

## Abstract

**Context:**

Sodium-glucose cotransporter 2 (SGLT2) inhibitors lower blood glucose levels by promoting urinary glucose excretion, but their overall effects on hormonal and metabolic status remain unclear.

**Objective:**

We here investigated the roles of insulin and glucagon in the regulation of glycemia in individuals treated with an SGLT2 inhibitor using mathematical model analysis.

**Methods:**

Hyperinsulinemic-euglycemic clamp and oral glucose tolerance tests were performed in 68 individuals with type 2 diabetes treated with the SGLT2 inhibitor dapagliflozin. Data previously obtained from such tests in 120 subjects with various levels of glucose tolerance and not treated with an SGLT2 inhibitor were examined as a control. Mathematical models of the feedback loops connecting glucose and insulin (GI model) or glucose, insulin, and glucagon (GIG model) were generated.

**Results:**

Analysis with the GI model revealed that the disposition index_/clearance_, which is defined as the product of insulin sensitivity and insulin secretion divided by the square of insulin clearance and represents the glucose-handling ability of insulin, was significantly correlated with glycemia in subjects not taking an SGLT2 inhibitor but not in those taking dapagliflozin. Analysis with the GIG model revealed that a metric defined as the product of glucagon sensitivity and glucagon secretion divided by glucagon clearance (designated production index_/clearance_) was significantly correlated with blood glucose level in subjects treated with dapagliflozin.

**Conclusion:**

Treatment with an SGLT2 inhibitor alters the relation between insulin effect and blood glucose concentration, and glucagon effect may account for variation in glycemia among individuals treated with such drugs.

In addition to their hypoglycemic action due to the promotion of urinary glucose excretion, sodium-glucose cotransporter 2 (SGLT2) inhibitors provide multiple clinical benefits, including prevention of heart failure and preservation of renal function [1-8]. Whereas such benefits are likely secondary effects of the inhibition of SGLT2 in proximal tubules, the hormonal and metabolic effects of SGLT2 inhibitors at the level of the whole body are not fully understood. These drugs augment endogenous glucose production (EGP) under specific conditions [9-12]. Although the mechanism underlying this phenomenon has remained unclear, it has been attributed to an increase in blood glucagon level reflecting either a compensatory response to urinary glucose loss [13] or direct stimulation of pancreatic α-cells [12]. A study based on pancreatic clamp analysis showed that the SGLT2 inhibitor–induced increase in EGP is, however, independent of the blood concentration of either glucagon or insulin [11].

Mathematical models are informative for the study of phenomena in which various factors interact with each other and individual effects cannot be assessed directly [14, 15], with one example being the regulation of glucose homeostasis. We previously developed models for the regulation of blood glucose concentration based on time series data for blood glucose and insulin levels during consecutive hyperglycemic and hyperinsulinemic-euglycemic clamp analyses [15, 16]. Using these models, we showed the relevance of insulin clearance to the regulation of glucose homeostasis [16]. Furthermore, we proposed a novel metric, termed disposition index_/clearance_ (DI_/cle_) and derived from insulin sensitivity, secretion, and clearance, and we showed that it well represents the capacity for glucose handling by insulin [16].

To gain insight into the effects of SGLT2 inhibitors on glucose metabolism, we have now mathematically analyzed hormonal and glycemia data obtained during a hyperinsulinemic-euglycemic clamp from individuals with type 2 diabetes under treatment with the SGLT2 inhibitor dapagliflozin. We here provide evidence that the effect of glucagon is a key determinant of interindividual variation in glycemia among individuals treated with dapagliflozin.

## Methods

### Study Participants and Measurements

This study was conducted in accordance with the Declaration of Helsinki and its amendments was approved by the Ethics Committee of Kobe University Hospital (approval number: C180035), and it was registered in the Japan Registry of Clinical Trials (clinical trial number: jRCTs051180058). The participants were individuals with type 2 diabetes who were aged 20 to 75 years and had not taken an SGLT2 inhibitor within the past 1 month. They were recruited at Kobe University Hospital between August 2017 and September 2020. All study participants provided written consent. Exclusion criteria are shown in Supplementary Table S1 [17].

Dapagliflozin (Forxiga; AstraZeneca K.K., Osaka, Japan–Ono Pharmaceutical Co. Ltd., Osaka, Japan) was administered to the study participants at a dose of 5 mg/day, and a 75-g oral glucose tolerance test (OGTT) and a hyperinsulinemic-euglycemic clamp test were performed on separate days between 3 and 6 days after the initiation of dapagliflozin treatment. The clamp test was conducted with the use of an artificial endocrine pancreas as described previously [18]. Detailed information for the OGTT and clamp test is provided in Supplementary Text S1 [17], and data obtained from 68 participants were analyzed (Supplementary Fig. S1 [17]).

For a control cohort not treated with SGLT2 inhibitors, we used previously published data obtained for an OGTT and a clamp test in 120 individuals comprising 50, 18, and 52 subjects with normal glucose tolerance, impaired glucose tolerance, or type 2 diabetes, respectively [15]. The study was approved by the Ethics Committee of Kobe University Hospital, and written informed consent was obtained from all participants. In this earlier study of ours, a total of 121 participants, who had been recruited from October 2008 to June 2014 at Kobe University Hospital, were initially included. Among these 121 individuals, the data for blood glucose levels at 120 minutes after the initiation of OGTT (PG120) was omitted for 1 subject. Therefore, we excluded this subject, resulting in the current study having 120 subjects. Detailed information on all subjects is provided in Tables 1 and 2.

**Table 1.** Characteristics of subjects with or without SGLT2 inhibitor treatment

Characteristic	With SGLT2i treatment	Without SGLT2i treatment			
		Total	NGT	IGT	T2DM
Number	68	120	50	18	52
Sex (male/female)	46/22	66/54	23/27	11/7	32/20
Age (years)	60.5 (54.0-69.0)	40.0 (28.0-58.0)	27.5 (25.0-34.0)	38.0 (33.0-42.5)	58.5 (43.3-63.8)
BMI (kg/m^2^)	25.5 (23.0-29.1)	23.7 (20.1-27.1)	20.4 (18.9-22.4)	25.0 (20.9-31.6)	25.1 (23.1-28.7)

Data for age and BMI are presented as median (25-75% interquartile range). Abbreviations: BMI, body mass index; IGT, impaired glucose tolerance; NGT, normal glucose tolerance; SGLT2i, sodium-glucose cotransporter 2 inhibitor; T2DM, type 2 diabetes mellitus.

**Table 2.** Oral glucose tolerance test and glucose clamp data for study subjects with or without SGLT2i treatment

Parameter	With SGLT2i treatment	Without SGLT2i treatment			
		Total	NGT	IGT	T2DM
OGTT-derived parameters					
FPG (mmol/L)	6.08 (5.22-7.15)	5.11 (4.61-5.72)	4.77 (4.47-5.08)	5.11 (4.41-5.59)	5.74 (5.13-6.42)
2-h PG (mmol/L)	12.13 (10.10-14.99)	8.77 (6.44-14.15)	6.13 (5.72-6.95)	8.66 (9.10-9.99)	15.01 (11.39-17.51)
F-IRI (pmol/L)	48.5 (31.7-69.5)	41.7 (27.8-63.9)	35.8 (29.0-57.3)	66.7 (45.5-85.4)	41.7 (27.8-62.5)
Insulinogenic index	0.20 (0.11-0.41)	0.39 (0.18-0.74)	0.72 (0.47-1.25)	0.49 (0.33-0.83)	0.16 (0.08-0.28)
Matsuda Index	5.09 (2.95-6.88)	5.54 (3.90-8.20)	6.61 (5.44-8.76)	3.43 (2.40-4.65)	5.19 (3.61-8.83)
UGER (mg kg^−1^ min^−1^)	1.38 ± 0.62				
Clamp-derived parameters					
GIR (mg kg^−1^ min^−1^)	4.17 ± 1.56	8.40 ± 2.87	10.37 ± 2.24	7.65 ± 2.69	6.79 ± 2.30
UGER (mg kg^−1^ min^−1^)	0.68 ± 0.32				
TGUR (mg kg^−1^ min^−1^)	3.49 ± 1.55				
Plasma glucose at steady state (mmol/L)	5.08 (4.72-5.44)	4.72 (4.33-5.22)	4.61 (4.11-4.94)	4.83 (4.25-5.16)	4.47 (4.88-5.37)
Serum insulin at steady state (pmol/L)	262.5 (194.8-467.1)	754.9 (636.9-923.7)	680.6 (566.5-779.6)	924.4 (791.7-1135.9)	826.5 (715.3-944.5)
ISI (×10^4^)	3.24 (1.90-4.60)	9.21 (6.05-12.03)	12.8 (10.05-16.10)	6.65 (3.73-10.10)	6.45 (4.49-9.28)

Data are presented as median (25-75% interquartile range) or as mean ± SD. Insulin sensitivity index (ISI) corresponds to 100 × TGUR [mg kg−1 min−1]/(plasma glucose level at the end of the clamp [mg/dL]/serum insulin level at the end of the clamp [μU/mL]) in SGLT2i-treated subjects, or to 100 × GIR [mg kg−1 min−1]/(plasma glucose level at the end of the clamp [mg/dL]/serum insulin level at the end of the clamp [μU/mL]) in cases without SGLT2i treatment.

Abbreviations: 2-h PG, plasma glucose level at 2 hours after glucose ingestion; F-IRI, fasting serum immunoreactive insulin level; FPG, fasting plasma glucose level; GIR, glucose infusion rate at steady state; IGT, impaired glucose tolerance; NGT, normal glucose tolerance; OGTT, oral glucose tolerance test; SGLT2i, sodium-glucose cotransporter 2 inhibitor; T2DM, type 2 diabetes mellitus; TGUR, tissue glucose uptake rate (GIR minus UGER); UGER, urinary glucose excretion rate.

### Mathematical Models

A mathematical model of the feedback loop that connects glucose and insulin, termed the glucose-insulin (GI) model, was developed. This model, which includes glucose flux from blood to urine, is an extension of a model we previously proposed [16]. SGLT2 inhibitors are antidiabetic drugs with the unique pharmacological characteristics of promoting urinary glucose excretion to nonphysiological levels. We therefore measured the urinary glucose excretion of the study participants and included this parameter in our model. A mathematical model of the feedback loop that connects glucose, insulin, and glucagon, termed the glucose-insulin-glucagon (GIG) model, was also generated. These models were fitted to the clamp test, as described previously [16]. Detailed descriptions of these models are provided in Supplementary Text S2 [17].

### Parameter Estimation

The parameters of the models (with the exception of $k_{8}$ and $k_{u}$) for reproduction of the normalized time course of the clamp test were estimated by a meta-evolutionary programming method to search the minimum globally and subsequent application of the nonlinear least squares technique to search the minimum locally, as described previously [15], for each of the 68 patients with type 2 diabetes treated with the SGLT2 inhibitor (Supplementary Text S2 [17]). Each parameter of the GI model was estimated in the range from $10^{-4}$ to $10^{4}$. Each parameter (with the exception of $k_{\mathrm{ratio}}$) of the GIG model was estimated in the range from $10^{-4}$ to $10^{6}$. The parameter $k_{\mathrm{ratio}}$ was estimated in the range from $10^{-10}$ to 1 because it represents the molar ratio of posthepatic insulin to C-peptide. The parameters were estimated to minimize the objective function value, which is defined as the residual sum of the square (RSS) between the actual time course obtained by clamp analysis and the model trajectories. RSS in the GI model (RSS_GI) is given by the following equation:


$$\begin{array}{rl} \mathrm{RSS}\_\mathrm{GI}\;= & \frac{n_{I}}{n_{G_{c}}+n_{I}}\overset{n_{G_{c}}}{\underset{i=1}{\sum }}\;[G_{c}(t_{i})-G_{\mathrm{csim}}(t_{i}){]}^{2}\\ & +\frac{n_{G_{c}}}{n_{G_{c}}+n_{I}}\overset{n_{I}}{\underset{i=1}{\sum }}\;[I(t_{i})-I_{\mathrm{sim}}(t_{i}){]}^{2} \end{array}$$

RSS in the GIG model (RSS_GIG) is given by the following equation:


$$\begin{array}{rl} 3\mathrm{RSS}\_\mathrm{GIG}\;= & \frac{n_{I}+n_{G_{g}}+n_{C_{P}}}{n_{G_{c}}+n_{I}+n_{G_{g}}+n_{C_{P}}}\sum_{i=1}^{n_{G_{c}}}{\left[G_{c}(t_{i})-G_{\mathrm{csim}}(t_{i})\right]}^{2}\\ & +\frac{n_{G_{g}}+n_{C_{P}}+n_{G_{c}}}{n_{G_{c}}+n_{I}+n_{G_{g}}+n_{C_{P}}}\sum_{i=1}^{n_{I}}{\left[I(t_{i})-I_{\mathrm{sim}}(t_{i})\right]}^{2}\\ & +\frac{n_{C_{P}}+n_{G_{c}}+n_{I}}{n_{G_{c}}+n_{I}+n_{G_{g}}+n_{C_{P}}}\sum_{i=1}^{n_{Gg}}{\left[G_{g}(t_{i})-G_{\mathrm{gsim}}(t_{i})\right]}^{2}\\ & +\frac{n_{G_{c}}+n_{I}+n_{G_{g}}}{n_{G_{c}}+n_{I}+n_{G_{g}}+n_{C_{P}}}\sum_{i=1}^{n_{C_{P}}}{\left[C_{P}(t_{i})-C_{\mathrm{psim}}(t_{i})\right]}^{2} \end{array}$$

where $n_{G_{c}}$, $n_{I}$, $n_{G_{g}}$, and $n_{C_{P}}\;$ are the total numbers of time points for measurement of blood glucose, insulin, glucagon, and C-peptide, respectively, in the hyperinsulinemic-euglycemic clamp, $t_{i}$ is the time of the *i*th time point, $G_{c}(t)$ is the time-averaged blood glucose concentration within the time range (t−5) min to *t* min for each 1-minute interval, I(t) is the blood insulin concentration at *t* min, $G_{g}(t)$ is the blood glucagon concentration at *t* minutes, and $C_{P}(t)$ is the blood C-peptide concentration at *t* minutes. $G_{\mathrm{csim}}(t)$, $I_{\mathrm{sim}}(t)$, $G_{g\mathrm{sim}}(t)$, and $C_{\mathrm{psim}}(t)$ are simulated blood glucose, insulin, glucagon, and C-peptide concentrations. Blood glucose, insulin, glucagon, and C-peptide concentrations of each subject were normalized by the corresponding maximum values. The numbers of parents and generations in the meta-evolutionary programming were 400 and 4000, respectively.

The parameters $k_{8}$ and $k_{u}$ are the same and are calculated by the following equation:


$$[\mathrm{Urinary}\;\mathrm{Glucose}]=\int_{0}^{120}\;k_{8}G_{c}\mathrm{BV}dt$$

where BV denotes blood volume, as described in Supplementary Text S2 [17].

### Statistical Analysis

Reduced models for plasma glucose, as well as serum insulin and glucagon concentrations (Supplementary Tables S2 and S3 [17]), were evaluated according to the Akaike information criterion (AIC) [19]. The sum *N* of the number of data points for each variable measured in the experiment was the same for compared models. AIC was therefore calculated as follows:


AIC=Nlog(RSS)+2K

where *K* is the number of estimated parameters of the model. The association between indices was evaluated with Spearman's correlation test or standardized major axis regression [20].

## Results

### Mathematical Model for Plasma Glucose and Serum Insulin Concentrations: GI Model

To gain insight into the effect of SGLT2 inhibitors on whole-body glucose homeostasis, we performed a hyperinsulinemic-euglycemic clamp test and an OGTT in individuals with type 2 diabetes who are treated with dapagliflozin. A flow diagram for the recruitment of and the baseline characteristics of the study subjects as well as the parameters directly obtained during the 2 tests are shown in Supplementary Fig. S1 [17] and Tables 1 and 2, respectively.

**Figure 1.**
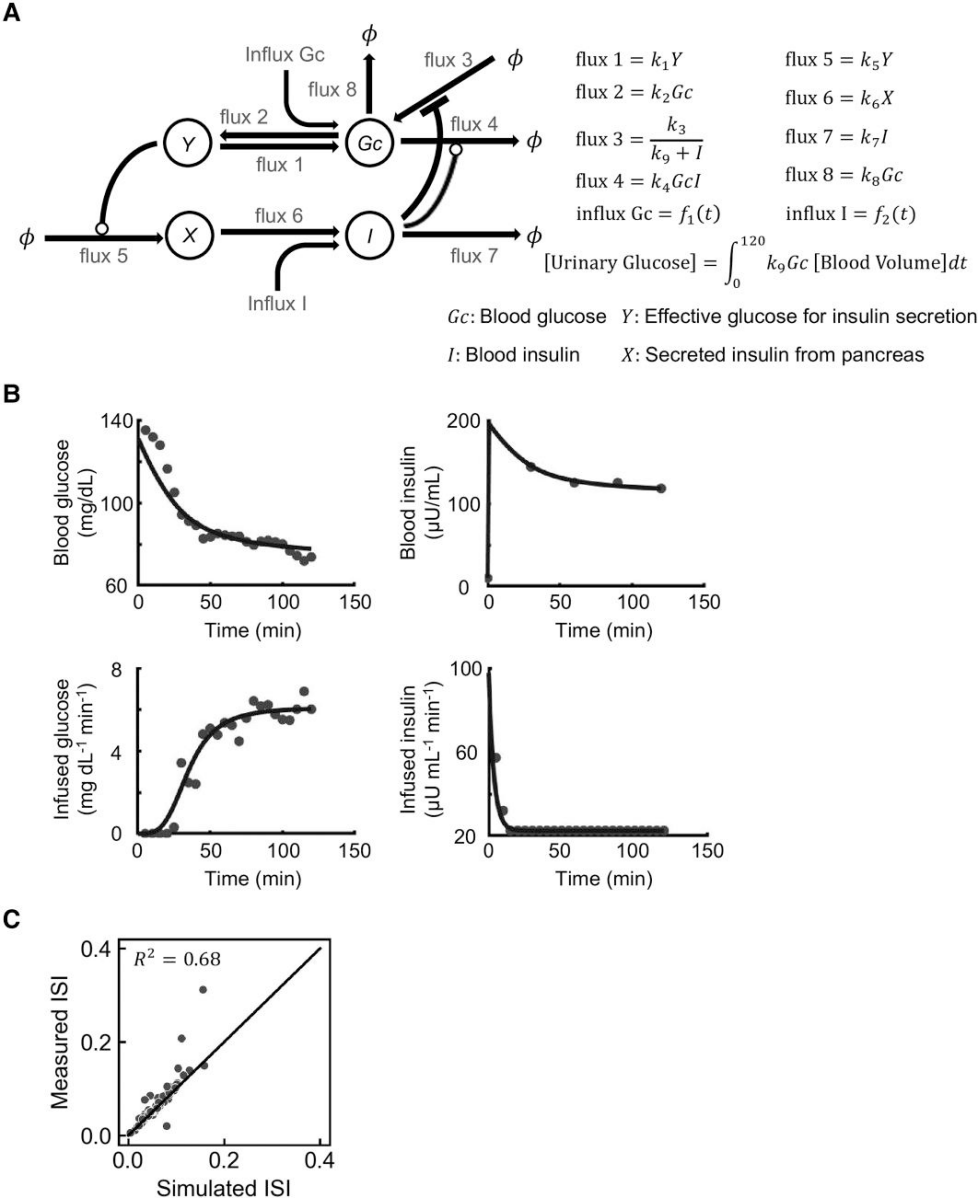
Mathematical model of the feedback loop that links glucose and insulin (GI model). A, Model diagram. The letters in the circles indicate variables of the model, the arrows indicate fluxes, and the lines ending in circles or bars indicate activation and inactivation, respectively. ϕ indicates a fixed value. The variables *Gc* and I represent blood glucose and insulin concentrations, respectively, and the variable *Y* corresponds to the effective glucose concentration for induction of variable *X*, which represents insulin secreted from pancreatic β-cells. Functions $f_{1}(t)$ and $f_{2}(t)$ are described in Supplementary Text S2 [17]. B, Time courses of blood glucose and insulin concentrations as well as of glucose and insulin infusion during the hyperinsulinemic-euglycemic clamp for study participant #5. Lines indicate the simulation, and black circles indicate the time course data from the experiment. Simulated values for each variable were rescaled to absolute concentration and plotted. C, Scatter plot for simulated vs measured insulin sensitivity index (ISI). Each point corresponds to the values for a single subject. Measured and simulated ISI values were calculated from the normalized time course and plotted. $R^{2}$ is the coefficient of determination.

We analyzed the time series data of plasma glucose and serum insulin levels during the clamp test with a mathematical model. We generated the model by adding the flux of glucose from blood to urine to a model that we proposed previously [15] (Fig. 1A, Supplementary Text S2 [17]). This new model was designated the *GI model*, given that it is based on the interaction of these 2 components. We estimated the parameters of the GI model for each study participant in order to reproduce the time course of the clamp test. The simulated and measured data for one subject are shown in Fig. 1B. To confirm that the simulation appropriately reflects the characteristics of the study subjects, we evaluated the consistency between the insulin sensitivity index (ISI) calculated from the actual measurements and that calculated from the simulation data, as described previously [15]. The coefficient of determination ($R^{2}$) between measured ISI and simulated ISI was 0.68 (Fig. 1C), suggesting that the model captures the essential characteristics of the time series data.

### Relation Between Estimated Parameters in the GI Model and Blood Glucose Level

We recently proposed a new insulin-related metric designated the disposition index_/clearance_ (DI_/cle_), which is defined as the product of insulin sensitivity and insulin secretion divided by the square of insulin clearance [16]. The DI_/cle_ can be considered as a metric that reflects adjustment of the disposition index (generally represented by the product of insulin sensitivity and insulin secretion) for insulin clearance, and it serves as a good indicator of the ability of insulin to regulate blood glucose concentration in individuals with various levels of glucose tolerance [16].

The relation between DI_/cle_ and blood glucose level is given by the following equation:


$$\mathrm{PG}120\;∼\;\frac{k_{4}k_{5}}{{k_{7}}^{2}}=DI_{/\mathrm{cle}}$$

The indices $k_{4}$, $k_{5}$, and $k_{7}$ correspond to insulin sensitivity, insulin secretion, and insulin clearance, respectively, and PG120 to the plasma glucose level at 120 minutes after initiation of the OGTT [16]. The coefficients of $k_{4}$, $k_{5}$, and $k_{7}$ in DI/cle were determined in the previous study. Given that our previous cohort was not treated with SGLT2 inhibitors [16], we examined whether this relation also holds in the present cohort treated with dapagliflozin.

**Figure 2.**
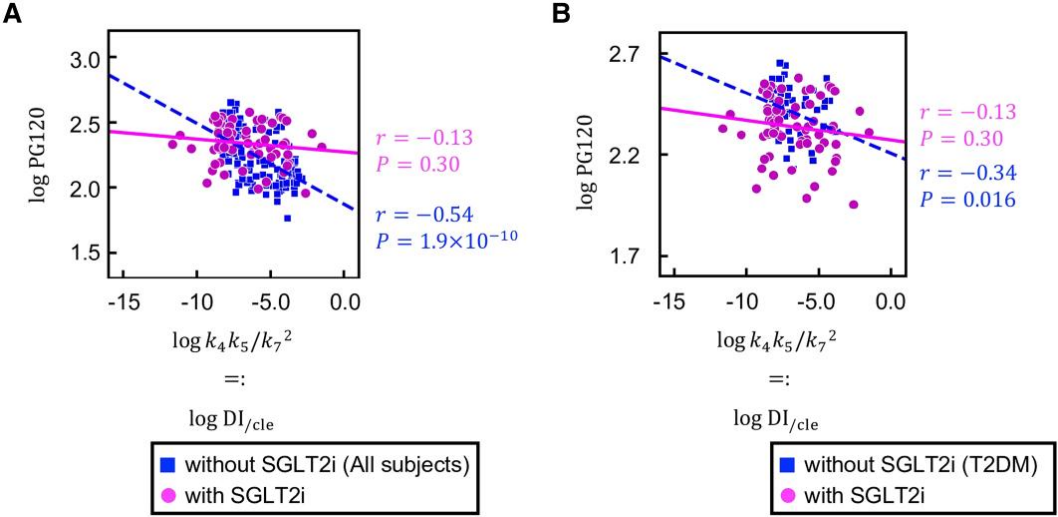
Relation between DI_/cle_ and blood glucose concentration. A, Scatter plot and fitted linear regression lines for $\log\;k_{4}k_{5}/k_{7}^{2}$ and logPG120. Each point corresponds to the values for a single subject. Blue squares and magenta circles indicate subjects not treated or treated with a sodium-glucose cotransporter 2 inhibitor (SGLT2i), respectively. Blue dotted and magenta solid lines indicate the corresponding linear regression lines. B, Scatter plot and fitted linear regression lines for $\log\;k_{4}k_{5}/k_{7}^{2}$ and logPG120. Blue squares indicate subjects with type 2 diabetes who are not treated with an SGLT2i, and magenta circles are as in (A). *r* is Spearman's correlation coefficient, and the *P* values are for testing the hypothesis of no correlation. The indices $k_{4}$, $k_{5}$, and $k_{7}$ correspond to insulin sensitivity, insulin secretion, and insulin clearance, respectively. PG120 is plasma glucose concentration at 120 minutes during the oral glucose tolerance test.

Given that insulin lowers blood glucose levels, it is assumed that a high insulin sensitivity, elevated insulin secretion, or diminished insulin clearance will lead to a decrease in blood glucose concentration. There would thus be expected to be a negative correlation between DI_/cle_ and blood glucose levels. As expected, in our previous cohort, DI_/cle_ ($k_{4}k_{5}$/ $k_{7}^{2}$) was negatively correlated with PG120 (r=−0.54,$P=5.6\times 10^{-10}$) (Fig. 2A), supporting the notion that DI_/cle_ represents the ability of insulin to regulate blood glucose concentration [16]. Given that the indices $k_{4}$, $k_{5}$, and $k_{7}$ in the GI model correspond to the same insulin-related parameters as in our previous model, DI_/cle_ for the present cohort is also given as $k_{4}k_{5}$ /$k_{7}^{2}$ in the GI model. In contrast to the previous cohort, however, DI_/cle_ in the current cohort was not significantly correlated with PG120 (r=−0.13,P=0.30) (Fig. 2A). The slopes of the regression lines for the 2 groups of subjects were significantly different (P<0.01). DI_/cle_ and PG120 also showed a significant correlation for the 49 subjects from previous cohort who have type 2 diabetes (r=−0.34, P=0.016). (Fig. 2B). The difference in the slopes of the regression lines for the type subjects with type 2 diabetes in the previous and present cohorts did not achieve statistical significance (P=0.06). These results suggested that DI_/cle_ is not sufficient to explain the interindividual variation in blood glucose levels among individuals with type 2 diabetes who are treated with an SGLT2 inhibitor.

### Production Index_/Clearance_ Is Positively Correlated With Blood Glucose Level Under SGLT2 Inhibitor Treatment

Given that DI_/cle_ did not adequately capture individual glycemic variation in subjects treated with an SGLT2 inhibitor, we next examined the contribution of glucagon to glycemic regulation in the present cohort. The serum concentration of glucagon during the OGTT was not significantly correlated with glycemia (Supplementary Fig. S2 [17]), consistent with a previous finding that blood glucagon level is independent of the increased EGP in subjects treated with SGLT2 inhibitors [11]. We therefore attempted to mathematically evaluate the overall effect of glucagon. By analogy with DI_/cle_, we hypothesized that the overall effect of glucagon is increased as glucagon sensitivity and the ratio of glucagon secretion to glucagon clearance are increased (Supplementary Text S3 [17]). We termed the product of these 2 parameters the production index/clearance (PI_/cle_):


$$\begin{array}{c} PI_{/\mathrm{cle}}=G_{\mathrm{gsen}}G_{\mathrm{gsec}/\mathrm{cle}} \end{array}$$

where $G_{\mathrm{gsen}}$ and $G_{\mathrm{gsec}/\mathrm{cle}}$ indicate glucagon sensitivity and the ratio of glucagon secretion to glucagon clearance, respectively. The product of $G_{\mathrm{gsen}}\;$ and $G_{\mathrm{gsec}/\mathrm{cle}}$ was calculated mathematically by the following equation (Supplementary Text S3 [17]):


(1) $$\begin{array}{c} G_{\mathrm{gsen}}G_{\mathrm{gsec}/\mathrm{cle}}=\frac{G_{g0}G_{0}f_{g}}{G_{\mathrm{ss}}I_{\mathrm{ss}}} \end{array}$$

where $G_{g0}$, $G_{0}$, $G_{\mathrm{ss}}$, $I_{\mathrm{ss}}$, and $f_{g}$ indicate blood glucagon level in the fasting state, blood glucose levels in the fasting state and at the steady state during insulin administration, blood insulin level at the steady state during insulin administration, and infused glucose at the steady state during the hyperinsulinemic-euglycemic clamp, respectively.

**Figure 3.**
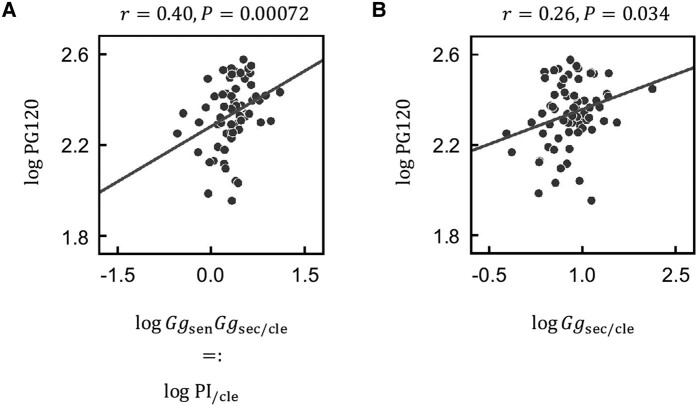
Relation between the estimated glucagon-related parameter PI_/cle_ and blood glucose concentration. Scatter plots for $\log\;G_{\mathrm{gsen}}G_{\mathrm{gsec}/\mathrm{cle}}$ vs logPG120 (A) and for $\log\;G_{\mathrm{gsec}/\mathrm{cle}}$ vs logPG120 (B) are shown. $G_{\mathrm{gsen}}$ and $G_{\mathrm{gsec}/\mathrm{cle}}$ correspond to glucagon sensitivity and the ratio of glucagon secretion to glucagon clearance, respectively. Each point corresponds to the values for a single subject of the present cohort. *r* is Spearman's correlation coefficient, and the *P* values are for testing the hypothesis of no correlation. PG120 represents plasma glucose concentration at 120 minutes during the oral glucose tolerance test.

PI_/cle_ calculated with Eq (1) was significantly correlated with PG120 in the cohort of the present study (r=0.40, P=0.00072) (Fig. 3A), suggesting that the effect of glucagon likely explains at least in part the interindividual variation in glycemia under SGLT2 inhibitor treatment. The numerator of $G_{\mathrm{gsen}}$ includes blood glucose level in the fasting state ($G_{0}$) (Supplementary Text S3 [17]). However, $G_{\mathrm{gsec}/\mathrm{cle}}$, which does not contain a glycemia-related index in its numerator, was also significantly correlated with PG120 (r=0.26, P=0.034) (Fig. 3B). These results suggested that the correlation between $G_{\mathrm{gsen}}G_{\mathrm{gsec}/\mathrm{cle}}$ and PG120 was not simply attributable to the fact that $G_{\mathrm{gsen}}G_{\mathrm{gsec}/\mathrm{cle}}$ contains a glycemia-related index in its numerator.

### Mathematical Model for Blood Glucose, Insulin, and Glucagon Concentrations (GIG Model) Also Shows a Positive Association Between PI_/cle_ and Blood Glucose Level

Given that PI_/cle_ estimated from fasting and steady-state parameters in Eq (1) was correlated with PG120 in the present cohort, we further analyzed the contribution of glucagon to glycemic regulation by mathematical model analysis. We developed a mathematical model of the feedback loop that links glucose, insulin, and glucagon with the use of time series data obtained from the study participants during the hyperinsulinemic-euglycemic clamp (Supplementary Text S4, Supplementary Figs. S3 and S4, Supplementary Tables S2 and S3 [17]). We designated this model the *GIG model*.

In this model, the parameters $k_{\mathrm{GN}}$, $k_{\mathrm{GgS}}$, and $k_{\mathrm{GgC}}$ conceptually correspond to glucagon sensitivity, glucagon secretion, and glucagon clearance, respectively (Supplementary Fig. S3 [17]). By analogy to DI_/cle_ ([insulin sensitivity × insulin secretion]/[insulin clearance]^2^), $k_{\mathrm{GN}}k_{\mathrm{GgS}}/k_{\mathrm{GgC}}^{2}$ is considered as the numerically estimated PI_/cle_. We calculated these parameters from the model estimated to be optimal for the greatest number of subjects out of 54 types of mathematical model based on AIC (Supplementary Text S5, Supplementary Tables S2 and S3 [17]).

Given that glucagon increases blood glucose concentration, a high glucagon sensitivity, enhanced glucagon secretion, and diminished glucagon clearance would be expected to result in an increase in blood glucose level, and $k_{\mathrm{GN}}k_{\mathrm{GgS}}/k_{\mathrm{GgC}}^{2}$ would therefore be expected to be positively correlated with the blood glucose concentration. Analytically defined PI_/cle_ calculated with Eq (1) as $G_{\mathrm{gsen}}G_{\mathrm{gsec}/\mathrm{cle}}$ and numerically defined PI_/cle_ determined by the mathematical model as $k_{\mathrm{GN}}k_{\mathrm{GgS}}/k_{\mathrm{GgC}}^{2}$ were significantly correlated (Supplementary Fig. S5A [17]). Furthermore, $k_{\mathrm{GN}}k_{\mathrm{GgS}}/k_{\mathrm{GgC}}^{2}$ was significantly correlated with PG120 (Supplementary Fig. S5B [17]). These results collectively suggested that the effect of glucagon contributes to interindividual variation in blood glucose levels among individuals with type 2 diabetes who are treated with an SGLT2 inhibitor. Similar correlations between numerically defined PI_/cle_ by the mathematical model and $G_{\mathrm{gsen}}G_{\mathrm{gsec}/\mathrm{cle}}$ or PG120 were apparent when the coefficient of $k_{\mathrm{GgC}}$ was set at 1 or 3 ($k_{\mathrm{GN}}k_{\mathrm{GgS}}/k_{\mathrm{GgC}}$ or $k_{\mathrm{GN}}k_{\mathrm{GgS}}/k_{\mathrm{GgC}}^{3}$) (Supplementary Fig. S5C–S5F [17]). Of note, the half-life of insulin calculated using GI model and that calculated using GIG model showed significant correlation (Supplementary Fig. S6 [17], suggesting the validity of these models.

## Discussion

Given that SGLT2 inhibitors promote urinary glucose excretion independently of insulin action, these drugs likely affect the relation between insulin effect and blood glucose concentration. We recently showed that DI_/cle_, a metric derived from insulin sensitivity, secretion, and clearance, represents the ability of insulin to regulate glycemia in individuals not taking an SGLT2 inhibitor. We have now shown that the relation between DI_/cle_ and glycemia does not hold for individuals treated with an SGLT2 inhibitor, underscoring the influence of such drugs on the relation between insulin effect and blood glucose level. We further showed that, among individuals with a relatively high DI_/cle_, the corresponding PG120 value was greater for those not taking than for those taking an SGLT2 inhibitor. Conversely, when DI_/cle_ was relatively low, the opposite phenomenon was observed. DI_/cle_ might therefore serve as a predictive factor for the effect of SGLT2 inhibitors on blood glucose level. Given that the glucose-lowering effect of SGLT2 inhibitors varies among individuals [21-23] and that the reason for this variation is not fully understood, it will be of interest to investigate whether DI_/cle_ indeed proves informative for prediction of the clinical effect of SGLT2 inhibitors.

By analogy to DI_/cle_, we have now developed a glucagon-related metric, PI_/cle_, derived from glucagon sensitivity, secretion, and clearance, and validated its effect in 2 different models. PI_/cle_ estimated by 2 different methods ($G_{\mathrm{gsen}}G_{\mathrm{gsec}/\mathrm{cle}}$ and $k_{\mathrm{GN}}k_{\mathrm{GgS}}/k_{\mathrm{GgC}}^{2}$) correlated with blood glucose concentration in individuals treated with dapagliflozin. These results, together with the insufficiency of DI_/cle_ to capture blood glucose levels in such individuals, suggest that glucagon action plays an important role in determining blood glucose concentration in patients treated with SGLT2 inhibitors. Evidence suggests that SGLT2 inhibitor treatment is associated with enhancement of EGP [9, 11] and that blood levels of glucagon, which increases EGP physiologically, do not explain this phenomenon [11]. We showed that PI_/cle_, but not blood glucagon concentration, correlates with blood glucose level, suggesting that blood glucagon concentration may not adequately represent the effect of the hormone in the body, and that PI_/cle_ might serve as a metric for evaluation of the overall effect of glucagon.

The present study has several limitations. First, we investigated subjects within 1 week of initiation of SGLT2 inhibitor administration. Our results might therefore differ from those obtained after long-term drug administration, as suggested by a previous study [24]. Future studies need to include a broader population, including individuals who have been administered SGLT2 inhibitor treatment for varying lengths of time and those who have not been administered SGLT2 inhibitors. Second, the study subjects were Japanese individuals with a relatively low body weight. It remains unclear whether our findings can be generalized to individuals of different ethnicities and body compositions. Third, given that we used blood parameters obtained during an OGTT and hyperinsulinemic-euglycemic clamp, it is unclear to what extent PI_/cle_ examined in the present study reflects the dynamics of glucagon in the body. Although this study only investigated the correlation between the overall effect of glucagon on glucose (PI/cle) and PG120 under SGLT2 inhibitor administration, it may not be possible to separately identify glucagon secretion and clearance in our model. Since the equations in this study are relatively simple and approximate only for a relatively narrow range of plasma molecular concentrations, more detailed and accurate equations for various concentration ranges need to be studied in the future. Assessment of glucagon sensitivity, secretion, and clearance with more accuracy and parameter identifiability will require studies that examine the effects of exogenous glucagon administration, ideally with a clamp test involving continuous glucagon infusion. Moreover, glucagon clearance has been postulated to contribute to the uncertain relation between glucagon concentration and glucagon secretion [12, 13, 25], likely because both blood glucose and glucagon concentrations are expected to rise when glucagon clearance is reduced. Further studies are therefore required to understand the relation between glucagon sensitivity, glucagon secretion, and glucagon clearance.

In conclusion, with the use of mathematical model analyses, we have shown that DI_/cle_ cannot fully account for interindividual variation in blood glucose levels among individuals with type 2 diabetes who are treated with an SGLT2 inhibitor, whereas PI_/cle_ is able to do so, at least in part. Further studies are required to provide a better understanding of the relative roles of insulin and glucagon in the regulation of blood glucose concentration.

## Data Availability

All datasets generated during and analyzed during the current study are not publicly available but are available from the corresponding author on reasonable request.

## Clinical Trial Information

Clinical Trial Registration number jRCTs051180058.

## Abbreviations

AIC: Akaike information criterion
DI/cle: disposition index/clearance ([insulin sensitivity × insulin secretion]/[insulin clearance]^2^)
EGP: endogenous glucose production
GI model: model of feedback loop connecting glucose and insulin
GIG model: model of the feedback loop connecting glucose, insulin, and glucagon
ISI: insulin sensitivity index
OGTT: 75-g oral glucose tolerance test
PG120: blood glucose levels at 120 minutes after the initiation of OGTT
PI/cle: production index/clearance
RSS: residual sum of the square
SGLT2: sodium-glucose cotransporter 2
